# Introgression from farmed escapees affects the full life cycle of wild Atlantic salmon

**DOI:** 10.1126/sciadv.abj3397

**Published:** 2021-12-22

**Authors:** Geir H. Bolstad, Sten Karlsson, Ingerid J. Hagen, Peder Fiske, Kurt Urdal, Harald Sægrov, Bjørn Florø-Larsen, Vegard P. Sollien, Gunnel Østborg, Ola H. Diserud, Arne J. Jensen, Kjetil Hindar

**Affiliations:** 1 Norwegian Institute for Nature Research (NINA), NO-7485 Trondheim, Norway.; 2 Rådgivende Biologer, NO-5059 Bergen, Norway.; 3 Norwegian Veterinary Institute, NO-7485 Trondheim, Norway.

## Abstract

After a half a century of salmon farming, we have yet to understand how the influx of genes from farmed escapees affects the full life history of Atlantic salmon (*Salmo salar* L.) in the wild. Using scale samples of over 6900 wild adult salmon from 105 rivers, we document that increased farmed genetic ancestry is associated with increased growth throughout life and a younger age at both seaward migration and sexual maturity. There was large among-population variation in the effects of introgression. Most saliently, the increased growth at sea following introgression declined with the population’s average growth potential. Variation at two major-effect loci associated with age at maturity was little affected by farmed genetic ancestry and could not explain the observed phenotypic effects of introgression. Our study provides knowledge crucial for predicting the ecological and evolutionary consequences of increased aquaculture production worldwide.

## Teaser

Early and late life history and growth are affected by farmed salmon introgression across 105 wild populations of Atlantic salmon.

## INTRODUCTION

Aquaculture increases worldwide in both production volume and number of fish species (*1*). Escapees from fish farms pose a threat to wild stocks. Outside their native range, escapees contribute to spread of alien species (*2*). Inside their range, escapees mate with wild conspecifics (*3*), thus potentially alienizing local populations through genetic changes.

Both artificial and natural selection during domestication lead to genetic changes. Interbreeding between farmed escapees and wild conspecifics thereby induces a genetic load and contributes to maladaptation of the wild population. Theoretical models predict that such interbreeding decreases population fitness and viability (*4*–*9*), but our knowledge of the ecological impact of farmed fish escaping and breeding in the wild is limited.

The Atlantic salmon (*Salmo salar* L.) has emerged as an important model system for understanding farmed to wild genetic introgression (*10*). The farmed Atlantic salmon has been selected for c. 13 generations to enhance economically important traits, foremost growth, but also age at maturity, filet color, disease resistance, and several other traits (*11*–*13*).

Since the startup of aquaculture in the 1970s, the number of farmed Atlantic salmon has increased to outnumber wild salmon 1000-fold (*14*). In Norway, farmed genetic introgression has been quantified in 239 wild populations comprising most of the national salmon stock [(*15*); see also (*3*)]. In the 68 (28%) most severely affected populations, introgression ranged from 10% to more than 50% estimated average genetic ancestry to farmed salmon. No genetic changes were found in only 80 (33%) of the populations. At present, farmed escapees are evaluated as the greatest threat to wild salmon in Norway (*16*). This is alarming as Norway holds about one-third of the total European Atlantic salmon stock (*14*). Although less studied outside Norway, interbreeding between farmed and wild Atlantic salmon occurs in many parts of its natural range on both sides of the Atlantic (*17*–*20*).

A particular concern following gene flow from farmed salmon are genetic changes in life-history traits, as these are closely connected to fitness and demography. The Atlantic salmon shares its life cycle between freshwater and seawater and undergoes two major physiological changes associated with life-history decisions (*21*). Eggs laid in autumn hatch in rivers in spring where juveniles usually spend one to four years before undergoing a developmental adaptation to salt water, called smoltification, and migrate to sea for foraging. After leaving the river, Atlantic salmon spend between one and three winters at sea, and sometimes more, before maturation and returning to their natal river to reproduce. As the farmed salmon is strongly selected for faster growth and thereby increased developmental speed, the timing of both life-history decisions may be altered by farmed genetic introgression. Age at maturity in Atlantic salmon is controlled by a few major-effect loci (*22*–*24*), potentially underlying an effect of farmed introgression on life history.

Farmed-wild genetic interactions are well studied in Atlantic salmon through experimental studies, most of them in laboratory or seminatural enclosures and a handful in controlled rivers (*25*–*29*). In 2017, a review of these studies by Glover *et al.* (*10*) concluded that gene flow between farmed and wild salmon most likely leads to changes in life-history traits and reduced population growth, and that we lack documentation of these changes in wild populations.

Documenting biological changes following farmed salmon introgression in wild populations is difficult. A main challenge is that molecular estimates of farmed genetic ancestry of an individual fish are uncertain because of the close genetic relatedness between farmed and wild fish; therefore, we only expect to detect an effect of introgression when the biological signal is strong (fig. S1) (*30*). Despite methodological challenges, studies have now documented decreased egg size when controlling for body size (*31*) and reduced juvenile survival (*32*, *33*) following introgression. We have previously documented changes in the number of years spent at sea (sea age) and increased size at maturity within sea age across a large number of populations (*30*). However, a large-scale analysis of the effect of introgression on life history and growth throughout the life cycle, and how this varies across natural populations, is warranted.

Here, we used growth patterns in the scales from 6926 adult salmon collected in 105 rivers from 58^o^ to 69^o^ north, mainly sampled between 2010 and 2017, to infer how introgression affects freshwater and seawater growth, age-specific probability of seaward migration, and age at maturity. We tested for among-population variation in the effect of introgression by including the average smolt age and average sea age of each population as linear covariates in the models and evaluated, using the Akaike information criterion (AIC), whether an interaction between each of these terms and farmed genetic ancestry improved the model fit. We also tested the effect of introgression on the allele frequency at two major-effect loci [the *vgll3_TOP_* and *six6_TOP_* single-nucleotide polymorphisms (SNPs); see the work by Barson *et al.* (*22*)] and whether variation at these loci could explain the observed effects of introgression on life history and growth. We relate the empirical findings to pace-of-life (POL) theory.

## RESULTS

### Smolt age and freshwater growth

**Fig. 1.**
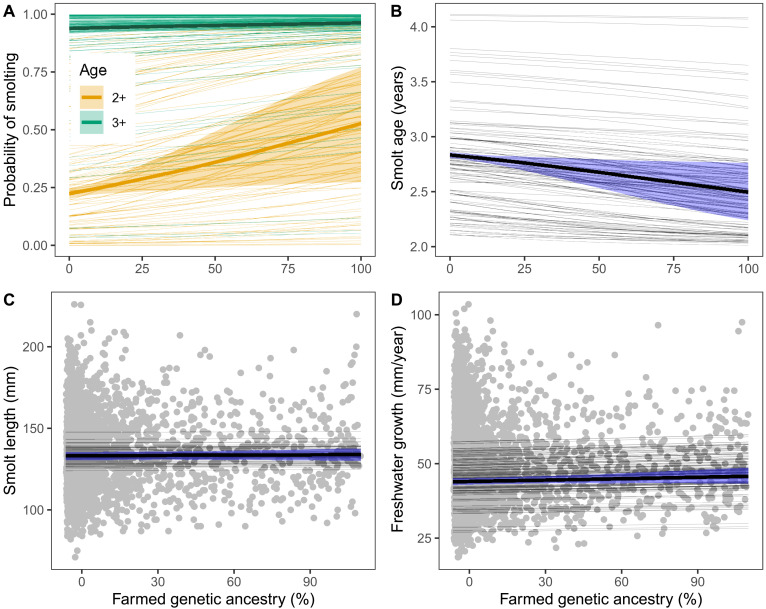
Effect of introgression (farmed genetic ancestry) on early life history. Figure panels show effects on different traits: (**A**) probability of smolting, (**B**) smolt age, (**C**) smolt length, and (**D**) freshwater growth. Thick lines show average effects across populations and shaded areas the associated 95% CI. Thin lines show population-specific effects. Parameter estimates are given in table S2.

Age and growth of salmonid fishes can be inferred from growth patterns in their scales. To estimate the effect of introgression on smolt age, we used a mixed-effects binomial regression model with probability of smoltifying as the response variable and population and year as random effects. On average, the probability of smolting at two years of age more than doubled [133%, 95% confidence interval (CI): 22 to 252%] because of introgression, from a 22% probability in genetically wild fish to a 50% probability in genetically farmed fish (Fig. 1A, thick orange line). At three years of age, the estimated increase was much weaker (2.6%, 95% CI: −1.1 to 5.0%), with a 95% CI overlapping zero (Fig. 1A, thick green line).

Our model predicted large among-population variation in the effect of introgression on probability of smolting at two years of age (Fig. 1A, thin orange lines). The populations ranged from having no effect to more than a fourfold increase in the probability of smolting from genetically wild to genetically farmed fish. The among-population variation arises for two reasons. First, variation in the intercept among populations generates among-population differences in the effect of introgression due to the nonlinear relationship between the logit scale, the scale on which the regression is linear, and the probability scale. On the probability scale, the regression has zero and one as asymptotes, and, in the absence of interaction terms, the effect is therefore strongest at probability 0.5. Second, there were some statistical support for among-population variation in the effect of introgression in the form of a positive interaction term between average sea age and farmed genetic ancestry, improving the model by −0.3 AIC units (table S1). This suggests that the effect of introgression increased with average sea age.

The changes in probability of smolting in the average population led to an expected decrease in smolt age of 0.34 years (95% CI: 0.07 to 0.59 years) from an expected smolt age of 2.83 years in genetically wild fish to 2.49 years in genetically farmed fish (Fig. 1B, thick line). Among populations, the predicted effect of introgression ranged from no change to a decrease of 0.61 years (Fig. 1B, thin lines).

Using the growth pattern in the scale, we back-calculated the smolt length by assuming isometry between length growth in the scale and the body. From this, we could calculate the average annual freshwater growth in body length. Smolt length and freshwater growth were analyzed using mixed-effects models with population and year as random effects.

We found little, if any, effect of farmed genetic ancestry on smolt length (Fig. 1C). The change from genetically wild to genetically farmed fish was estimated at only 0.5% (95% CI: −2.0 to 2.9%). This suggests that the decrease in smolt age was due to an increased growth, which is expected as most of the genetic variance in probability of smolting is explained by genetic variance in size (*34*). With a 0.5% increase in size and an estimated 12.0% decrease in smolt age, the annual growth would need to be increased by 14.2% in genetically farmed fish. In the empirical measures of back-calculated growth, however, the increase was estimated at only 4.3% (95% CI: −1.1 to 10.1%) (Fig. 1D). This discrepancy illustrates that our method gives conservative estimates of biological changes following introgression, despite our attempts to correct this bias (see Materials and Methods). We found no evidence for variation among populations in the effect of introgression on smolt length or on freshwater growth (table S1).

### Age and size at maturity and growth at sea

**Fig. 2.**
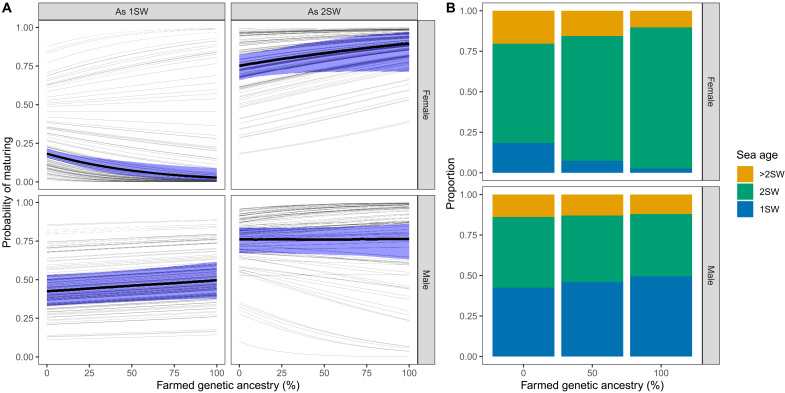
Effect of introgression on probability of maturing as 1SW or 2SW for the two sexes. (**A**) Thick lines show average effects across populations and shaded areas the associated 95% CI. Thin lines show population-specific effects. Parameter estimates are given in table S3. (**B**) The resulting distribution of sea ages from the average effects across populations.

To quantify the effect of farmed introgression on age at maturity, we first estimated the change in probability of maturing at different sea ages (numbers of winters at sea) in a generalized mixed-effects model with river and year as random effects. In this model, and the models below, we included data source (whether the samples were obtained through recreational, broodstock, or scientific fishing) as a fixed factor. Because sexes differ genetically in life history at sea (*22*), we estimated effect of introgression separately for the two sexes. The effect of farmed genetic ancestry on probability of maturing differed across sex and sea age (Fig. 2A).

For females in the average population, the probability of maturing as a one-sea-winter salmon (1SW) decreased by a factor of 6.9 (95% CI: 1.9 to 27.6) from 18% in genetically wild to less than 3% in genetically farmed fish (Fig. 2A, top left, thick line). However, the effect of introgression varied among populations. There was strong evidence for a negative interaction between farmed genetic ancestry and population average sea age improving the model by −2.4 AIC units (table S1). This interaction generated positive effect of introgression in populations with high probability of maturing as 1SW and negative for populations with lower probability (Fig. 2A, top left, thin lines). In contrast, there was a universal positive effect of farmed genetic ancestry on the probability of maturing as 2SW and no evidence for an interaction with population average sea age in the model (Fig. 2A, top right, and table S1). In the average population, the probability of maturing as 2SW increased by a factor of 1.2 (95% CI: 1.0 to 1.4) from 75% in genetically wild to 90% in genetically farmed fish (Fig. 2A, top right, thick line). Because of the nonlinear link function in the model, the effect of introgression was predicted to be strongest around a probability of maturing of 50% (i.e., in populations with high sea age). Combining the average effects for probability of maturing as 1SW and 2SW results in a substantial increase of 2SW females with increasing farmed genetic ancestry (Fig. 2B).

For males in the average population, the probability of maturing as 1SW was estimated to increase by a factor of 1.2 (95% CI: 1.0 to 1.4) from 42% in genetically wild to 50% in genetically farmed fish (Fig. 2A, bottom left, thick line). There was no evidence for among-population variation in this relationship (table S1). Furthermore, there was no evidence for an effect of introgression on probability of maturing as 2SW in the average population (Fig. 2A, bottom right, thick line), but there was some evidence for an interaction with population average smolt age. Including this interaction improved the model by −1.4 AIC units (table S1) and led to a predicted positive effect of introgression on male probability of maturing as 2SW in populations with high smolt age [indicating cold rivers (*35*)] and a predicted negative effect of introgression in populations with low smolt age (indicating warm rivers). Among populations, the predicted effect of introgression ranged from an increase of 11% (from 76 to 87%) to a decrease of 31% (from 55 to 24%) in probability of maturing as 2SW between genetically wild and farmed fish (Fig. 2A, bottom right, thin lines).

**Fig. 3.**
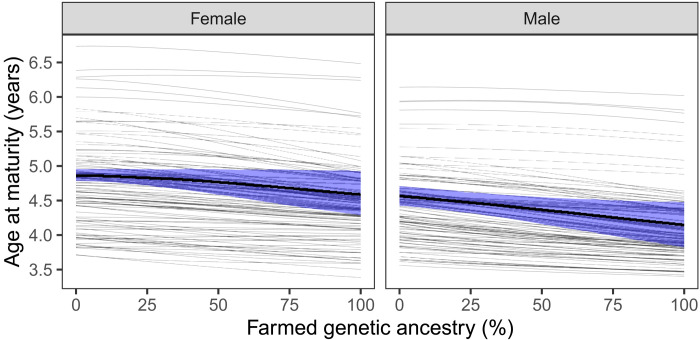
Effect of introgression on age at maturity for the two sexes. Thick lines show average effects across populations and shaded areas the associated 95% CI. Thin lines show population-specific effects.

By combining the analyses on probability of smolting and maturing, we obtained estimates of the effect of introgression on age at maturity (Fig. 3). In both sexes, the genetically farmed fish were younger at maturity than genetically wild fish: 0.29 (95% CI: −0.03 to 0.60) years younger for females and 0.43 (95% CI: 0.11 to 0.73) years younger for males. Our model predicted among-population variation in the effect of introgression on age at maturity, ranging from no change to a decrease of 0.81 years in females and decrease of 0.67 years in males. While the average effect was weaker, the among-population variation was larger in females than in males.

The decrease in age at maturity was mainly caused by early life history (i.e., smolt age) and not number of years at sea. In the average population, the decrease in females was solely due to smolt age, while in males, smolt age contributed to 79% of the decrease.

**Fig. 4.**
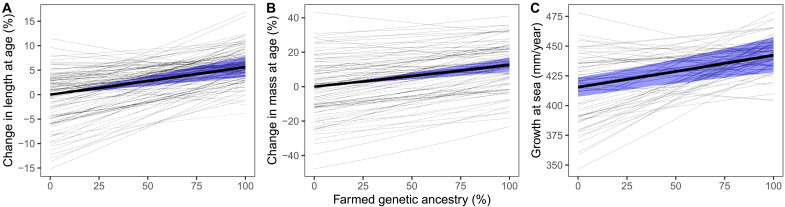
Effect of introgression on returning adult salmon. (**A**) Change in length, (**B**) change in mass, and (**C**) growth at sea. Thick lines show average effects across populations and shaded areas the associated 95% CI. Thin lines show population-specific effects. Parameter estimates are given in table S4.

To assess the effect of introgression on growth at sea, we first estimated the effect of introgression on length and mass of returning adult salmon before we analyzed the effect on back-calculated growth at different periods at sea using mixed-effects models. On average, the increase in size at sea age from genetically wild to genetically farmed salmon was estimated at 5.6% (95% CI: 3.6 to 7.7%) for length and at 12.7% (95% CI: 8.8 to 16.4%) for mass (Fig. 4, A and B). There was substantial among-population variation in these relationships. For both the model on length and the model on mass, including an interaction between farmed genetic ancestry and population average sea age substantially improved the model fit (by −5.9 AIC units for length and by −5.2 AIC units for mass; table S1). The effect of introgression on size was stronger and more positive in populations with lower sea age with a maximum of 20% increase in length and 38% increase in weight from genetically wild to genetically farmed fish, while in populations with high sea age, the effect became negative with a maximum decrease of 7% in length and 13% in mass from genetically wild to genetically farmed fish. These results suggest that the effect of introgression on size (Fig. 4, A and B) is less consistent across populations than the nearly universal negative effect on age (Fig. 3).

Because there was little effect of introgression on smolt length (Fig. 1C), the observed size increase of returns must be due to growth at sea. This is confirmed by our analysis on the effect of introgression on average annual lengthwise growth at sea (Fig. 4C). Growth increased by 6% (95% CI: 3 to 10%) from 415 mm/year in genetically wild salmon to 442 mm/year in genetically farmed fish (Fig. 4C). For growth at sea, there was also substantial among-population variation. Including an interaction between introgression and population average sea age and between introgression and average smolt age improved model fit by −3.7 AIC units (table S1). Both interactions were negative, but the interaction with sea age was stronger than that with smolt age. Hence, the effect of introgression on growth was most positive in populations with low sea age and most negative in populations with high sea age, with a weaker but qualitatively similar effect from low to high smolt age. This generated a pattern where populations with low growth potential were more positively influenced by introgression than populations with high growth potential (i.e., a negative correlation between intercept and slope). Across populations, the effect of introgression on annual growth varied from an increase of 28% to a decrease of 8% between genetically wild and genetically farmed fish (Fig. 4C, thin lines).

**Fig. 5.**
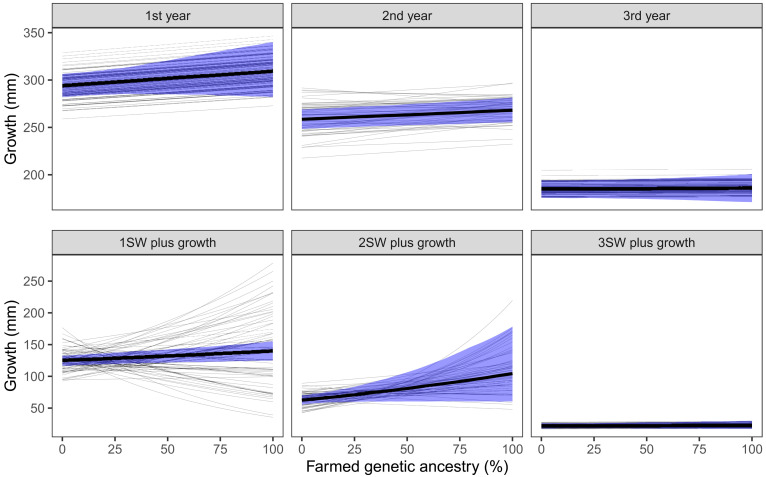
Effect of introgression on growth for different periods at sea. Thick lines show average effects across populations and shaded areas the associated 95% CI. Thin lines show population-specific effects. Parameter estimates are given in table S5.

To further understand the effect of introgression on growth at sea, we decomposed the total back-calculated growth into different periods at sea (Fig. 5). The periods are growth during the different years until end of the winter (as identified in the scales) and growth after winter during the return migration for the salmon that have started to mature. This latter growth is called plus growth. The strongest effect of introgression was on the plus growth for fish maturing as 1SW and 2SW, where genetically farmed fish grew 11.9% (95% CI: 1.9 to 22.5%) and 66.9% (95% CI: −2.3 to 179.0%) more than genetically wild, respectively. These were also the two growth periods with most among-population variation, ranging from negative effects of introgression on growth to very strong positive effects of introgression (Fig. 5, thin lines). The effect of introgression on plus growth was predicted to be strongest (most positive) in populations with low smolt and sea age (table S5).

For growth during the different years at sea, we also detected positive effects but less strong than for the plus growth and with less among-population variation (Fig. 5). The increase from genetically wild to genetically farmed fish was estimated at 5.8% (95% CI: −1.5 to 13.7%) and 3.7% (95% CI: 0.3 to 7.2%), for the first and second years at sea, respectively. We observed no effect of introgression on growth for the third year at sea or on the return migration of 3SW fish (Fig. 5).

### Major-effect loci

**Fig. 6.**
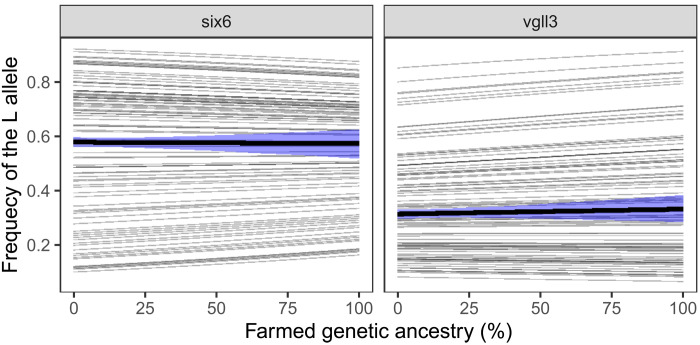
Effect of introgression on *vgll3_TOP_* and *six6_TOP_* L allele frequencies. The L allele is the allele associated with late maturation for each marker [see (*22*)]. Thick lines show average effects across populations and shaded areas the associated 95% CI. Thin lines show population-specific effects. Parameter estimates are given in table S6.

Recent discoveries of loci (the *vgll3_TOP_* and *six6_TOP_* SNPs) strongly associated with size and sea age at maturity (*22*) raise the question of whether the effect of introgression on life history and growth can be explained by allele frequency differences between wild and farmed salmon at these loci. We first tested whether we could find evidence for change in allele frequency with level of introgression. If farmed salmon are homogeneous with respect to allele frequency, then we would expect a negative interaction between wild-population allele frequency and farmed genetic ancestry. For *vgll3_TOP_*, we found no evidence of such interaction. Including an interaction term in the model increased the AIC by 0.9 units. For *six6_TOP_*, on the other hand, there was some evidence of a negative interaction (ΔAIC = −1.2). This suggests that the allele frequencies of *six6_TOP_* in the wild populations are homogenized with increasing farmed genetic ancestry. For neither SNPs did the allele frequency in the average population change with farmed genetic ancestry (Fig. 6, thick lines), in other words, the average allele frequency of all the genetically wild fish was estimated to be close to the average allele frequency of the genetically farmed fish.

To test the involvement of *vgll3_TOP_* and *six6_TOP_* in the effect of introgression on size and sea age at maturity, we compared the effect of introgression in models with and without these SNPs as fixed factors. Including *vgll3_TOP_* and *six6_TOP_* had almost no effect on the estimated slope (Table 1), except for the models with probability of maturing as 2SW as response (for both sexes). However, in all cases the difference in slope was smaller than the SE of the slopes and may therefore be due to sampling error.

**Table 1.** Effect of introgression (slope ± SE) on age and size at maturity when controlling or not for *vgll3_TOP_* and *six6_TOP_* genotypes as fixed factors in the model. Models not controlling for genotypes are the same as in the above analyses in Figs. 2A and 4. Estimates are based on the subset of data for which the two genotypes were available. In the responses, the probability of maturing as 1SW and 2SW is denoted *P*(1SW) and *P*(2SW), respectively. Units are given for each parameter, with “*a*” representing units of farmed genetic ancestry (*a* = 1 is 100% farmed genetic ancestry).

**Response**	**Not controlling**	**Controlling**	**Difference**	**Units**
*P*(1SW) female	−0.85 ± 0.51	−0.87 ± 0.51	0.02	log odds/*a*
*P*(1SW) male	−0.13 ± 0.23	−0.12 ± 0.24	−0.01	log odds/*a*
*P*(2SW) female	0.00 ± 0.35	−0.26 ± 0.42	0.26	log odds/*a*
*P*(2SW) male	−0.16 ± 0.37	−0.26 ± 0.42	0.10	log odds/*a*
Length at age	0.018 ± 0.007	0.020 ± 0.007	−0.002	ln(mm)/*a*
Mass at age	0.102 ± 0.026	0.101 ± 0.025	0.001	ln(g)/*a*
Average growth	0.043 ± 0.014	0.042 ± 0.014	0.001	ln(mm/year)/*a*

## DISCUSSION

Using an observational approach, we document that adult wild Atlantic salmon with genetic ancestry to farmed salmon matured at a younger age and grew faster than genetically wild fish. The reduced age at maturity was mostly due to changes in early (number of years in freshwater) rather than late life history (number of years at sea), while growth was increased in both life stages. We also observed large variation among populations in the effect of introgression.

### Benefits and limitations of the observational approach

Almost all previous studies on the effect of farmed genetic introgression have been by experimental manipulation (*10*). The observational approach that we have taken has both advantages and disadvantages compared to the experimental approach. An advantage of the observational approach is that we have measured the effects on individuals living in their natural environment. Hence, we have documented the changes as they have occurred in the real world and not through an experiment that, depending on its setup, will be more or less representative of the process in the wild. A second advantage is that we can study how the effects of introgression vary across many natural populations.

A disadvantage of the observational approach is that unobserved variables can create correlations between farmed introgression and phenotypic traits. However, because we compare fish within the same river the same year in our statistical models, we regard this problem to be limited in our case [see also discussion by Bolstad *et al.* (*30*)].

A second disadvantage is that we rely on scale material obtained from different sources. Our data come from anglers and broodstock fishing and a small amount from scientific fishing. This was necessary for obtaining a sufficient sample size, but the degree to which the data represent a random sample of the investigated populations can be questioned. Because of the high exploitation rate in Norway, we believe that fish caught by anglers provide a representative picture of the wild population, although not completely unbiased. We controlled for data type in the statistical analyses and did find systematic differences that were sometimes large (tables S3 to S6). However, while the intercept of our analysis can be biased because of sampling, it should not much affect the relationship with farmed genetic ancestry. In other words, while size or sea age of the salmon can affect its probability of being caught (*36*, *37*), it is more unlikely that this effect of size and sea age would vary strongly and systematically with farmed genetic ancestry.

A third disadvantage of our approach is that we rely on molecular markers for estimating farmed genetic ancestry. Because our measures of genetic ancestry include error, this leads to conservative estimates (attenuation) of the effect of farmed genetic introgression (*30*). Hence, we can only observe effects when the biological signal is strong, and it is therefore remarkable that effects are at all observed. To correct for the effect of attenuation, we have implemented bias correction (see Materials and Methods), but our estimates are still conservative.

Our study is based on salmon that survived and returned from the ocean and not the ones that died, making it difficult to separate effects of development and mortality. Mortality may have affected our estimates of average growth and effect of introgression if fast-growing fish systematically experience differential mortality from slow-growing fish. Mortality definitively affected average probability of smolting and maturing as fish delaying either of these transitions experience lower survival due to increased age. However, discerning the extent to which mortality also affected the observed differences between genetically farmed and genetically wild salmon is challenging. The use of only adult salmon in our study is not problematic per se, but it affects the trait definitions: the traits investigated in this study are probability of smolting and maturing, and growth given survival to maturity. The fact that survival is part of the trait definition is important to bear in mind when interpreting the results. Considering the advantages and shortcomings of the observational and experimental approaches, it is clear that a combination of observational and experimental studies is beneficial for attaining a best possible understanding of farmed introgression in the wild.

### Effects of introgression on growth and life history in Atlantic salmon

Previous experimental studies (*10*) paint the same picture of changes in early life history as our results from wild populations. Three studies performed under hatchery conditions find a higher smolting rate at early age for farmed fish (*38*–*40*). Results from experiments in the wild show no difference in the Burrishoole catchment experiment (*25*, *26*), but genetically farmed fish was found to be younger than genetically wild fish in both the River Imsa and the Guddalselva experiments (*27*, *28*). There is overwhelming evidence for increased freshwater growth for genetically farmed fish compared to genetically wild fish both under hatchery and wild conditions (*10*), which is also suggested by our results. However, the difference in growth between genetically farmed and wild fish is substantially reduced in the natural environment compared to hatchery conditions. The reduced difference in the wild can be attributed to both reduced realized growth and increased mortality of genetically fast-growing fish (*41*).

We observed increased growth of genetically farmed salmon at the subadult stage (i.e., after smoltification). This is well documented under hatchery conditions (*10*) and was also reported in the Guddalselva experiment (*28*) and in a study based on hatchery-produced smolt of Imsa salmon, farmed salmon, and reciprocal crosses (*42*). We find the largest effect of farmed introgression on the plus growth (i.e., during the return migration). This is not reported before, but farmed salmon is known to have a high growth in the equivalent period under captive conditions (*43*).

In line with our results, a younger age at maturity among the offspring of farmed salmon was found in the Imsa experiment (*27*). Here, farmed-by-wild offspring were, on average, 3.4 years old at sexual maturity and significantly younger than wild Imsa fish at 4.2 years. This was primarily a result of younger smolt age of farmed offspring but also influenced by poor survival of wild age 1+ smolts. In this experiment that lasted a whole generation, the limited number of seaward migrating smolt makes inferences of marine life history difficult. A later study in the Imsa found no difference in sea age at maturity between groups (*42*), again pointing to smolt age as the main cause of reduced age at maturity. A contrasting result was found in the Burrishoole experiment (*25*), where age at maturity was higher for farmed than wild salmon, and this was caused by farmed salmon staying, on average, 0.9 years longer at sea. However, in this comparison, an Irish wild population was compared with a farmed strain originating from Norwegian wild populations (*25*). Presumably, both the domestication process and the origin of the farmed strain influence the resulting differences in life history between genetically wild and genetically farmed salmon, which was noted by Fleming and Einum (*38*) for the freshwater phase and by Bolstad *et al.* (*30*) for the seawater phase.

Differences in effect of introgression across populations are caused by either the effect of the genetic background into which the introgression occurs or genotype-environment interactions. The estimated among-population variation in the effect of introgression differed among traits. There was stronger variation on growth at sea and size at return, where the effect of introgression was both positive and negative, than on age where the effect of introgression was never positive. For growth in freshwater, we found no evidence for among-population variation in the effect of introgression.

We evaluated systematic population variation along two dimensions of life-history variation in the populations: average sea age, which is positively correlated with river discharge (*44*), and average smolt age, which is negatively correlated with river temperature (*35*). Populations with low average sea age had a stronger positive effect of introgression on growth at sea and therefore size at age but less reduction in age at smolting and maturity compared to populations with high sea age. Populations with low average smolt age had a more negative effect of introgression on probability of maturing as 2SW for males but more positive effect of introgression on growth at sea. However, for growth at sea, population differences in average sea age were more important than differences in average smolt age. The combined effect of sea age and smolt age generated a pattern in which naturally slow-growing populations had a positive effect of introgression on growth at sea, while this effect was negative in naturally fast-growing populations.

Life-history variation in Atlantic salmon is strongly associated with two SNPs: one on chromosome 9 in the region of the *six6* gene (*six6_TOP_*) and one on chromosome 25 in the region of the *vgll3* gene (*vgll3_TOP_*) (*22*). Even if the farmed salmon is not selected for a change in allele frequency at these loci, we would expect that introgression homogenizes the wild populations toward the farmed allele frequency. We do observe a weak homogenizing effect of introgression on *six6_TOP_* but not on *vgll3_TOP_*. Lack of a strong effect may be due to weak statistical power or a counteracting effect of natural selection. These two markers are among those with the highest observed among-population differentiation (i.e., F_ST_ outliers) across the salmon genome (*22*), which can be considered evidence for strong selection promoting population divergence and local adaptation. Gene flow from farmed escapees at these two markers is therefore expected to be strongly counteracted by natural selection. As our data are on adult salmon, which must have survived and returned to the river, thus giving natural selection ample opportunity, it is perhaps expected that there is little effect of introgression on *six6_TOP_* and *vgll3_TOP_*. Because there is little effect of introgression on the allele frequency at these markers, we would not expect them to explain the observed effect of introgression on life history and growth. Our results are in alignment with this.

### The way forward

For Atlantic salmon, we now have detailed knowledge on the effect of introgression on life history and growth across a large number of natural populations. However, despite large sample sizes, our estimates are uncertain and conservative. Additional molecular markers more accurately resolving level of farmed genetic ancestry among wild born fish could improve this substantially. Studying populations where the pedigree is known from molecular analyses could also prove useful for this purpose.

We know that changes in life history have demographic consequences for the wild populations, but it is difficult to make a quantitative assessment of the impact on population dynamics based on our results alone. Obviously, a few introgressed individuals will not have a large population-level impact but at what point the level of introgression becomes biologically important at the population level is not easy to evaluate. Combining the empirical results on survival and life history with (stochastic) matrix population models could provide a more detailed understanding of the ecological effect of introgression. This would also help identifying at which point the level of introgression in the population becomes critical. Modeling studies have investigated this question (*7*, *8*), but more work is needed for making firm conclusions.

There are several hundred species in aquaculture, but we have little knowledge of introgression beyond Atlantic salmon (*45*). More empirical studies on other species are sorely needed to understand the global impact of aquaculture. In the meantime, the results from farmed salmon could potentially be generalized. One avenue toward a more general understanding is to study how introgression affects an organism’s pace of life (POL).

A major axis of life-history variation is captured by the “fast-slow” or POL continuum, with fast species characterized by early age at first reproduction and slow species by late age at first reproduction (*46*, *47*). While POL has traditionally been studied among species, it can also be studied among populations and individuals (*48*, *49*). We have provided solid evidence that the age at reproduction in Atlantic salmon decreases with introgression. It therefore seems that the selection for increased growth in aquaculture leads to genetic change toward a faster POL.

The POL syndrome (POLS) hypothesis has been suggested as an extension of the POL concept. According to this hypothesis, evolution along the POL continuum affects a whole suite of traits, with fast individuals or organisms typically being more aggressive and bolder with elevated metabolism and faster growth than slow individuals (*49*–*51*). In salmon, experiments have shown that farmed compared to wild fish have higher levels of aggression (*38*, *52*, *53*), more boldness in terms of shorter emergence time after exposure to artificial predator (*38*, *52*, *54*), increased dispersal (*42*, *55*), and decreased egg size when controlling for body size (*31*), all being traits associated with the fast end of POLS. Studies of gene transcription have shown that immune-related genes are down-regulated in farmed compared to wild salmon (*56*, *57*), while protein synthesis and metabolism are up-regulated in farmed compared to wild salmon (*56*, *58*), both supporting the POLS hypothesis. Wild and farmed salmon differ in allele frequency of structural variants (SVs) underlying behavioral traits during domestication, as well as SVs underlying immunity and metabolism (*59*). Collectively, the empirical evidence strongly supports our hypothesis that functional genetic differences between wild and farmed salmon can largely be explained by different positioning along the POLS axis.

Increased POL following farmed genetic introgression will have population level effects. Foremost, it will lead to maladaptation by offsetting important life history trade-offs and therefore lowered fitness of introgressed individuals. For example, selection for increased growth in farmed salmon has led to a higher susceptibility to predators (*60*), which can, at least partly, explain their observed lowered juvenile survival in the wild (*25*–*29*). At a general level, increased POL is also expected to increase the stochasticity of the population dynamics (*61*–*63*), which leads to a further reduction of the long-term population growth rate and population viability (*64*).

Genetic changes toward a faster POL may also affect the ecosystem. For example, changes in foraging behavior can have cascading effects. A recent study suggests that effects of intraspecific genetic and phenotypic variation can be equally important as species effects on community composition and ecosystem processes (*65*).

The evolution toward a faster POL during the domestication of Atlantic salmon is expected because of the strong selection for faster growth. Our study documents that these genetic changes are phenotypically expressed in wild Atlantic salmon with high farmed genetic ancestry. Other domesticated species, particularly those selected for fast growth, likely have similar genetic changes toward a faster POL, with predicted associated changes in life history and behavior. We therefore would expect strong demographic consequences of high gene flow from domesticated to wild conspecifics in many organisms. Actions to reduce genetic interaction between farmed and wild conspecifics are therefore of high importance.

## MATERIALS AND METHODS

### Experimental design

#### Data

The data comprise 6926 wild adult Atlantic salmon captured from 105 rivers in Norway, belonging to the Eastern Atlantic phylogenetic group (see table S7 for overview of rivers and sample sizes). The sampling was done between 1990 and 2017, with the bulk of the data (91%) after 2010 (fig. S2). Most data were collected either by recreational anglers during most of the run time in Norway or from fish collected for broodstock in the autumn and a smaller amount from scientific fishing, which aims at a random sample for estimating the proportion of escaped farmed salmon in the autumn. Because exploitation rates are high in Norway (40 to 60%), we believe that a random sample of fish caught by anglers is representative for the natural populations, although there might be biases in the data through fishing regulations and fishing gear. There is mixed evidence for nonrandom fishing by recreational anglers; most studies from Norway suggest a higher exploitation rate on 1SW salmon than on multi-SW salmon, whereas studies in Spain and the United Kingdom have shown higher exploitation rate on multi-SW salmon (*36*, *37*). A small subset of the angled fish had been selected for genetic analysis because of their large size in an earlier project; these were considered as representative for the wild population with regard to phenotypes expressed in freshwater but not for phenotypes expressed at sea. The rationale behind this is that smolt age and freshwater growth are only weakly correlated with sea age and size at return (smolt age versus sea age *r* = 0.01 among 6076 fish and freshwater growth versus size at return *r* = 0.06 among 3525 fish). In some rivers, broodstock sampling is known to avoid small salmon. On this background, we evaluated all broodstock sampling and excluded 455 fish in 24 rivers for the analyses on adult phenotypes. The percentage of nonrandom sampling in each river is given in table S7.

#### Phenotypic measurements

The angler or stocking personnel measured total length (millimeters; from the tip of the snout to the end of the caudal fin), evaluated and recorded the sex, and took a scale sample of each fish. Experienced scale readers analyzed the growth pattern in the scale and used this as a basis for excluding farmed salmon (*66*) (so that our data only consisted of wild-born salmon with various levels of farmed genetic ancestry), recording smolt and sea age, and measured the length between different life stages in the scale of each fish. Scale reading methods followed international guidelines on age and growth determination in salmon (*67*, *68*). Studies on marked and recaptured fish have found good correspondence between the period spent at sea versus sea age inferred using scale reading (*66*) and size at first capture versus back-calculated size (*69*, *70*).

We back-calculated length (in millimeters) of life stage *i* according to the equation *L_i_* = *L*_tot_*k_i_*, where *L*_tot_ is the total length of the fish and *k_i_* is the fraction of growth in length until life stage *i*. This fraction is *k_i_* = *S_i_*/*S*_tot_, where *S_i_* is the growth of the scale until life stage *i*, as measured across the criculi (growth rings) in the scale, and *S*_tot_ is the total radius of the scale. We measured growth in freshwater as *L_s_*/*a*, where the subscript *s* denotes life stage smolt and *a* is the corresponding age, while we measured growth at sea as *L_i_* − *L*_*i* − 1_, where the subscript *i* − 1 denotes the previous life stage. Hence, growth at sea is measured in units of smolt lengths. For all continuous traits, we removed individuals deviating more than four standard deviations from the mean before the analysis.

#### Large-effect loci

A subsample of our material, 3673 fish, was genotyped for two SNP markers. The two markers, *vgll3_TOP_* and *six6_TOP_*, are described by Barson *et al.* (*22*) and are both strongly associated with life history variation (*22*–*24*).

#### Farmed genetic ancestry

To estimate farmed genetic ancestry (level of introgression), we followed the exact same procedure as described by Bolstad *et al.* (*30*). This measure is based on 48 SNP markers that were selected for differentiation between wild and farmed salmon in Norway (*71*). The 48 diagnostic markers were identified by comparing a set of historical samples from 13 Norwegian wild salmon populations (samples from early- to mid-1980s) and farmed breeding kernels (samples from 1998 to 2008) in 4514 SNP markers. The SNPs showing the largest generic genetic differences between wild and farmed salmon were selected as diagnostic markers (*71*).

Because farmed to wild genetic introgression has occurred for many generations and from several genetically different breeding kernels, farmed ancestry was estimated in relation to one wild reference population and one farmed reference population (center points). The wild and farmed center points were *in silico* generated from a pool of genotypic data of historical samples (pre-aquaculture) from wild salmon populations and a pool of genotypic data of farmed salmon from the different breeding kernels. For each individual, the probability of belonging to the wild in silico population versus the farmed in silico population was estimated using STRUCTURE (*72*), as outlined by Karlsson *et al.* (*73*). This gave an estimate of the proportional ancestry (*P*_ind_) for each individual to the domesticated reference populations. The genetic ancestry to farmed fish was then calculated as (*P*_ind_ − 0.060)/(0.903 − 0.060), where 0.060 and 0.903 are the mean proportional ancestry in the wild and domesticated reference samples, respectively (*73*). This measure of farmed genetic ancestry is unbiased, so that a large sample of first-generation wild-farmed hybrids will have a mean value of 0.5, while a large sample of genetically wild and farmed individuals will have a mean value of 0 and 1, respectively (*73*). However, at the individual level, there will be large variation due to uncertainty in this measure (fig. S1) (*73*). The distribution of estimated farmed genetic ancestry of individual fish over rivers and sampling years is shown in fig. S2.

#### Correction for bias due to uncertainty in level of introgression

The uncertainty in level of introgression leads to an inflated range in the estimated level of introgression and strong downward bias in its effect on phenotypes [see the work by Bolstad *et al.* (*30*) and Hansen and Bartoszek (*74*), and fig. S1]. We therefore developed a method to correct for this bias. To do this, we first divided the data into two groups: individuals with a measured level of introgression zero or less in the first group and those above zero in the second group. The value zero is the mean introgression in the wild reference population. As the true level of farmed genetic ancestry cannot be negative, all individuals in the first group were assigned the value zero. The second group has an expected level of introgression between their estimated mean and the mean of all individuals. According to our simulations (see fig. S1), assigning these individuals the value of the weighted mean of these two means (weighted by the number of individuals underlying each mean) seemed to give the best bias correction. An unweighted mean would give an even more conservative bias correction. In the second step, we repeated the statistical analysis (see below) with these assigned values of level of introgression rather than the original estimates. We used the bias-corrected slope of this second analysis to calculate a correction factor, the ratio of the bias-corrected slope on the slope estimated on the original data. Last, we multiplied the correction factor with the original slope and its SE to get corrected values. Therefore, the correction did not alter the statistical significance of the results. In some cases, the correction factor was less than 1 or negative; this is not meaningful and represented cases where the relationship was highly uncertain. In these cases, the correction factor was ignored.

### Statistical analysis

#### Smolt age and freshwater growth

We analyzed the effect of introgression on smolt age using probability of smoltifying (*Y*) at a particular age (*a*) as a response variable in a mixed-effects binomial regression model with a logit link and assuming binomially distributed errors

$$\begin{array}{c} \text{ln}\frac{P(Y_{\mathrm{ijk}}=a)}{P(Y_{\mathrm{ijk}}>a)}=\mu +\beta_{1}(X_{\mathrm{ijk}}-X_{i••}+X_{•••})+\beta_{2}(A_{i••}-A_{•••})+\\ \beta_{3}(S_{i••}-S_{•••})+r_{i}+u_{\mathrm{ij}} \end{array}$$ (1)

where the subscripts *i*, *j*, and *k* denote population, smolting year, and individual, respectively; μ is the intercept; β_1_ is the within-population effect of introgression (*X*); β_2_ is the effect of average smolt age in each population (*A*); β_3_ is the effect of average sea age in each population (*S*); *r* is the random effect of river; *u* is the random effect of year nested within river; and the symbol • in the subscripts denotes that the variable is an average taken over the indicated levels. Hence, the term (*X_ijk_* − *X*_*i* • •_ + *X*_• • •_) represents the individual variation in farmed genetic ancestry once the among-population variation (*X*_*i* • •_) is removed. Average population smolt age and sea age were centered on their grand means, while the grand mean (*X*_• • •_) was added to the farmed genetic ancestry so that the intercept represents the probability of smoltifying for a fish without any farmed genetic ancestry in the average population. The rationale behind the within-population centering of *X* was that we wanted β_1_ to estimate the effect of farmed genetic ancestry within population. Both random effects were assumed to be Gaussian independent and identically distributed.

We fitted the model separately for smolt ages *a* = 2+ and *a* = 3+. In the *a* = 2+ model, the random effect of rivers was estimated to be zero and the term *r* was removed. The effect of introgression on smolt age can be obtained by combining these two models: age = 2*P*_2+_ + 3*P*_3+_(1 − *P*_2+_) + *A*(1 − *P*_3+_)(1 − *P*_2+_), where *P*_2+_ and *P*_3+_ are the probabilities of fish to smoltify at age 2+ and 3+, respectively, and *A* is the average age of the fish smoltifying older than 3+, *A* = 4.15 years in our data.

We analyzed freshwater growth and size in a mixed model with very similar structure as the one for probability of maturing, except that we used an identity link, the residuals were assumed to follow a Gaussian distribution, subscript *j* represents hatching year, and individual sea age was included as a fixed factor. The inclusion of sea age was to control for the large differences in adult size affecting the back-calculation if the assumption of isometry does not hold. For freshwater growth, we used back-calculated length growth in millimeters per year on the natural log scale [ln(mm/year)] as a response variable, while for smolt length, we used back-calculated length on the natural log scale (ln mm).

#### Age at maturity and growth at sea

We analyzed the effect of introgression on probability of maturing in a similar model to that of probability of smolting described above, but with *a* representing number of sea winters (*a* = 1SW or *a* = 2SW) at maturity and capture year (*u_j_*) as random effect. The model was fitted separately for males and females. In the female *a* = 1SW model, the random effect of river was estimated to be zero and the term *r* was removed.

We calculated age at maturity by first calculating sea age at maturity from the probabilities of maturing: sea age = *P*_1SW+_ + 2*P*_2SW+_(1 − *P*_1SW+_) + *A*(1 − *P*_1SW_)(1 − *P*_SW_), where *P*_1SW_ and *P*_2SW_ are the probabilities of fish to mature as 1SW and 2SW respectively, and *A* is the average sea age of the fish maturing after three or more winters at sea. In our data, *A* was 3.05 and 3.10 years for females and males, respectively. We then summed estimated smolt age with estimated sea age to get age at maturity.

The mixed model used for analyzing growth at sea and adult size at age was also similar to the one for analyzing probability of smolting, but with an identity link and Gaussian distributed residuals. In addition, we included Julian day (centered on day 200) as a covariate to control for day of capture. As response variable, we used the different measures of growth at sea as they are defined in the “Phenotypic measurements” section. The different growth and size measures were log-transformed and analyzed in separate models. For all models on probability of maturing, growth at sea, and size at return, we included data type (data from recreational, broodstock, or scientific fishing) as a fixed factor to control for differences in sampling regime.

#### Model selection

To test for evidence of variation across populations in the effect of introgression in all the above models, we evaluated interaction terms between the within-population variation in introgression (*X_ijk_* − *X*_*i* • •_ + *X*_• • •_) and population average smolt age or population average sea age (*A*_*i* • •_ − *A*_• • •_) using AIC (*75*). Using AIC, we also tested whether there was evidence for random variation in the effect of introgression (β_1_), by adding this as an additional random effect (technically a random regression model). If the model’s AIC value is reduced, then this means that the inclusion of the additional effect explains more variation in the data than what is expected at random. If the reduction in AIC is small (−2 < ΔAIC < 0), then the more complex model including the interaction is not much better in explaining the data than the simpler model without the interaction, while if the reduction is large (ΔAIC < −2), then the more complex model definitively is better.

#### Large-effect loci

To test the effect of introgression on allele frequency of large-effect loci (*six6_TOP_* and *vgll3_TOP_*), we fitted binomial mixed-effects model using a logit link, where the response variable *Y* was coded 0, 1, or 2, representing on the number of “L” alleles of each individual. [The L allele is the allele previously found to be associated with late maturation (*22*).] The statistical model was

$$\begin{array}{c} Y_{\mathrm{ijk}}\sim \text{Binomial}(2,p_{\mathrm{ijk}})\\ \text{ln}\frac{p_{\mathrm{ijk}}}{1-p_{\mathrm{ijk}}}=\mu +\beta_{1}(X_{\mathrm{ijk}}-X_{i••}+X_{•••})+\beta_{2}(p_{i••}-p_{•••})\\ +\beta_{3}(X_{\mathrm{ijk}}-X_{i••}+X_{•••})(p_{i••}-p_{•••})+r_{i}+u_{\mathrm{ij}}+v_{\mathrm{ijk}} \end{array}$$ (2)

where the subscripts *i*, *j*, and *k* denote population, capture year, and individual, respectively; μ is the intercept; β_1_ is the within-population effect of introgression (*X*); β_2_ is the effect of populations allele frequency (*p*); β_3_ is the interaction between introgression and allele frequency; *r* is the random effect of river; *u* is the random effect of year nested within river; *v* is an individual-level random effect to account for overdispersion; and the symbol • in the subscripts denotes that the variable is an average taken over the indicated levels. For the *six6_TOP_* model, there was no estimated variation across rivers and years, so the terms *r* and *u* were removed. For the *vgll3_TOP_* model, the variation in *r* and *v* was estimated to be zero and these terms were removed.

#### General

For all estimates, we obtained the 95% CIs by Monte Carlo simulation, assuming that the errors of the parameter estimates were multivariate normal with variance matrices equal to the estimated error variance matrices on their original scales. We performed all analyses in R (*76*) using the lme4 package for fitting the mixed models (*77*).

## Notes

View/request a protocol for this paper from *Bio-protocol*.
