# Supplementary material for: Introgression from farmed escapees affects the full life cycle of wild Atlantic salmon
Source: Sci Adv. 2021 Dec 22;7(52):eabj3397. doi: 10.1126/sciadv.abj3397 (PMC8694624; doi:10.1126/sciadv.abj3397)
Supplement: Supplementary file 1 — Figs. S1 and S2 Tables S1 to S7 [file sciadv.abj3397_sm.pdf]

Science Advances
AAAS

Supplementary Materials for

Introgression from farmed escapees affects the full life cycle of wild Atlantic salmon

Geir H. Bolstad*, Sten Karlsson, Ingerid J. Hagen, Peder Fiske, Kurt Urdal, Harald Sægrov, Bjørn Florø-Larsen, Vegard P. Sollien, Gunnel Østborg, Ola H. Diserud, Arne J. Jensen, Kjetil Hindar

*Corresponding author. Email: geir.bolstad@nina.no

Published 22 December 2021, *Sci. Adv.* **7**, eabj3397 (2021)
DOI: 10.1126/sciadv.abj3397

This PDF file includes:

Figs. S1 and S2
Tables S1 to S7

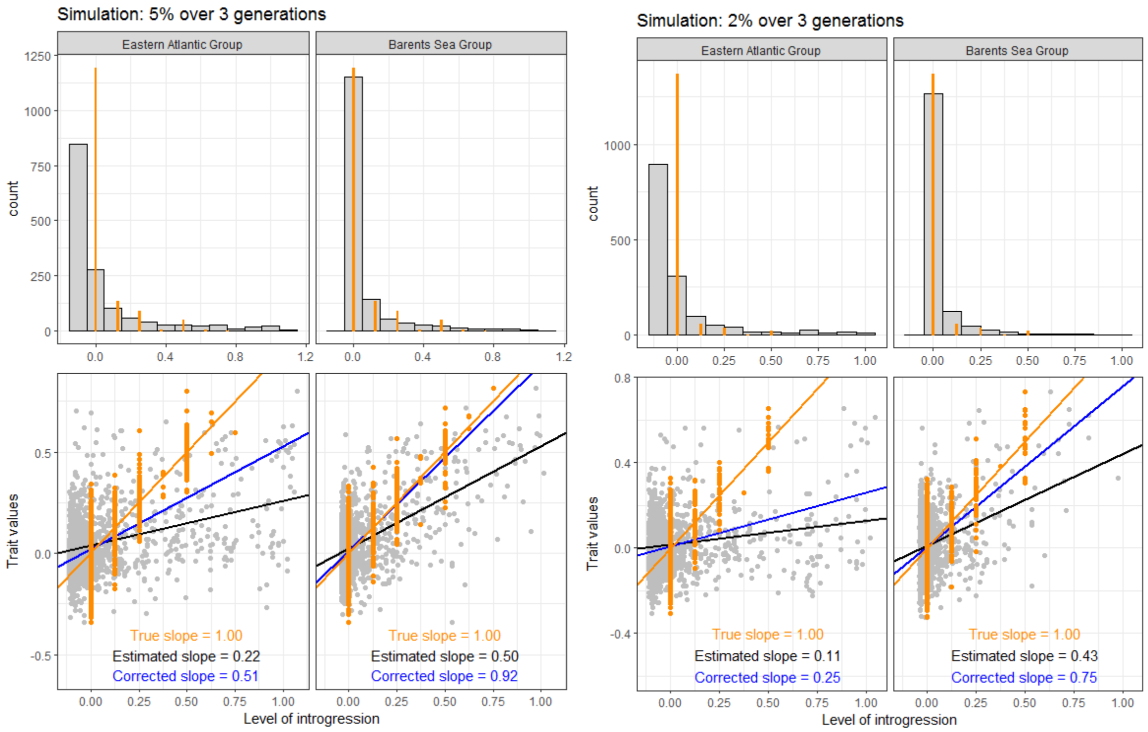

Figure S1. Evaluation of the bias correction method. Simulated data showing the effect of uncertainty in the level of introgression on the observed distribution of estimates (upper panel) and its estimated effect on trait values (lower panel). The true value of each individual and the true regression lines are shown in orange, while the estimated value is shown in grey and the estimated regression line in black. In blue, the bias corrected slope is given after the method described in the methods in the main text. Simulations shown for two different scenarios, one with a relatively high number of domesticated spawners (5% per year) in the population, and the other with relatively low (2%). Details of the simulation are given in the supplement of Bolstad et al. (*30*) under "Simulation of the effect of uncertainty in the estimated level of introgression".

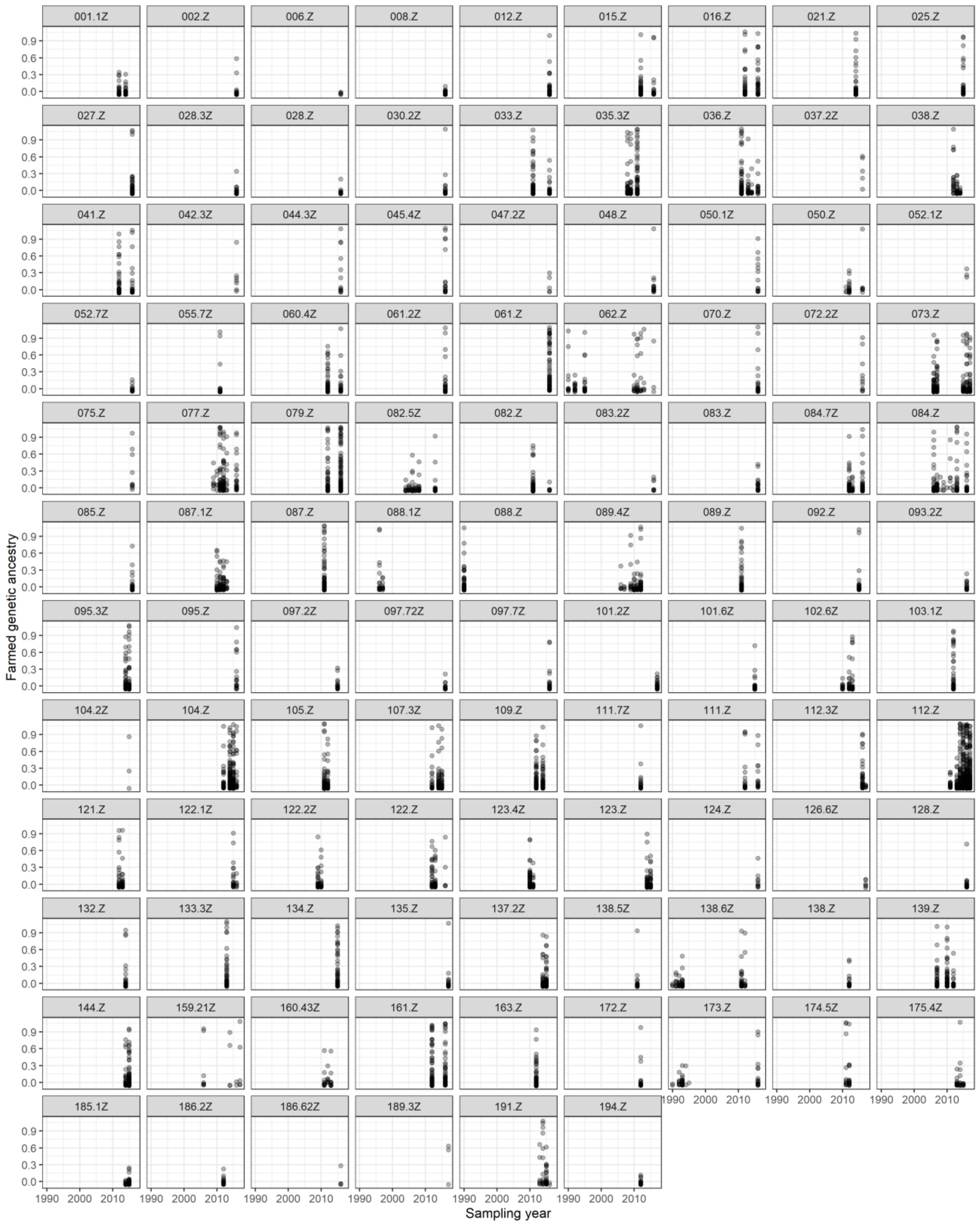

Figure S2. Distribution of estimated farmed genetic ancestry of individual fish over rivers and sampling years. See table S7 for river ID codes. The rivers are ordered from south to north.

Table S1. Model selection table. Change in Akaike's Information Criterion (ΔAIC) when the indicated interaction or randomly varying intercept is added to the model. Maximum likelihood (ML) was used to calculate ΔAIC for fixed effects, and restricted maximum likelihood (REML) for random effects.

Model	Interaction between farmed genetic ancestry and		Randomly varying effect of farmed genetic ancestry	ΔAIC
	**Average smolt age**	**Average sea age**		
Probability of smolting at 2+				
	X			1.1
		X		**-0.3**
			X	2.0
Probability of smolting at 3+				
	X			1.9
		X		1.7
			X	3.5
Back-calculated freshwater growth				
	X			2.0
		X		1.5
			X	2.0
Back-calculated freshwater size				
	X			2.0
		X		1.2
			X	3.4
Probability of maturing as 1SW for females				
	X			1.1
		X		**-2.4**
			X	2.0
Probability of maturing as 1SW for males				
	X			1.5
		X		2.0
			X	2.5
Probability of maturing as 2SW for females				
	X			0.2
		X		1.6
			X	1.9
Probability of maturing as 2SW for males				
	X			**-1.4**
		X		1.8
			X	3.0
Length at age				
	X			0.7
		X		**-5.9**
			X	0.5
Mass at age:				
	X			1.1
		X		**-5.2**
			X	3.5
Annual growth at sea				
	X			-1.6
		X		-3.5
	X	X		**-3.7**
			X	1.6
Growth first year at sea:				
	X			2.0
		X		1.9
			X	2.6

Model	Interaction between farmed genetic ancestry and		Randomly varying effect of farmed genetic ancestry	ΔAIC
	Average smolt age	Average sea age		
Growth second year at sea:				
	X			2.0
		X		**-2.1**
			X	3.0
Growth third year at sea:				
	X			1.7
		X		0.6
			X	2.6
Plus growth of 1SW:				
	X			-15.1
		X		-6.2
			X	-0.4
	X	X		**-16.1**
	X	X	X	-12.5
Plus growth of 2SW:				
	X			0.4
		X		**-0.5**
			X	4.0
Plus growth of 3SW:				
	X			1.8
		X		1.8
			X	3.9

Table S2. Parameter estimates for models on early life history. Estimate and associated standard error (SE) after correction for attenuation. The correction was done by multiplying the estimate and its standard error by *k*. Sample sizes are given by n. Units are given for each parameter, where "a" is farmed genetic ancestry (i.e. a = 1 is 100% farmed genetic ancestry).

Parameter	Units	Estimate	SE	*k*
Probability of smolting at 2+ (n = 6078):				
Intercept	log odds	-1.25	0.06	
Effect of population average smolt age	log odds/year	-4.06	0.17	
Effect of population average sea age	log odds/year	0.05	0.16	
Effect of introgression	log odds/a	1.35	0.58	4.13
Interaction: sea age and introgression	log odds/(year × a)	1.50	0.99	2.15
SD of intercept among years within river	log odds	0.64		
Probability of smolting at 3+ (n = 4072):				
Intercept	log odds	2.71	0.14	
Effect of population average smolt age	log odds/year	-3.92	0.26	
Effect of population average sea age	log odds/year	-0.01	0.28	
Effect of introgression (log odds/a)	log odds/a	0.51	0.36	1.79
SD of intercept among rivers	log odds	0.83		
SD of intercept among years within river	log odds	0.21		
Back-calculated freshwater growth (n = 3527):				
Intercept	ln(mm/year)	3.785	0.012	
Contrast 2SW	ln(mm/year)	0.122	0.009	
Contrast 3SW	ln(mm/year)	0.195	0.011	
Contrast 4SW	ln(mm/year)	0.321	0.029	
Contrast 5SW	ln(mm/year)	0.172	0.156	
Effect of population average smolt age	ln(mm/year)/year	-0.306	0.020	
Effect of population average sea age	ln(mm/year)/year	-0.040	0.031	
Effect of introgression	ln(mm/year)/a	0.036	0.023	1.91
SD of intercept among years within river	ln(mm/year)	0.163		
Back-calculated freshwater size (n = 3664):				
Intercept	ln mm	4.892	0.009	
Contrast 2SW	ln mm	0.013	0.007	
Contrast 3SW	ln mm	0.003	0.009	
Contrast 4SW	ln mm	0.014	0.023	
Contrast 5SW	ln mm	0.027	0.114	
Effect of population average smolt age	ln(mm)/year	0.009	0.014	
Effect of population average sea age	ln(mm)/year	0.065	0.021	
Effect of introgression	ln(mm)/a	0.004	0.011	1*
SD of intercept among rivers	ln mm	0.041		
SD of intercept among years within river	ln mm	0.054		

* The estimated correction factor was negative and therefore ignored (see methods).

Table S3. Parameter estimates for models on probability on maturing. Estimates and associated standard error (SE) after correction for attenuation. The correction was done by multiplying the estimate and its standard error by *k*. Sample sizes are given by n. Units are given for each parameter, where "a" is farmed genetic ancestry (i.e. a = 1 is 100% farmed genetic ancestry).

Parameter	Units	Estimate	SE	*k*
Probability of maturing as 1SW for females (n = 2760):				
Intercept	log odds	-1.49	0.11	
Contrast to fish caught for broodstock	log odds	-1.33	0.31	
Contrast to fish caught in scientific fishing	log odds	0.02	0.20	
Effect of population average smolt age	log odds/year	0.00	0.14	
Effect of population average sea age	log odds/year	-3.58	0.22	
Effect of introgression (log odds/a)	log odds/a	-2.12	0.67	1.95
Interaction: sea age and introgression	log odds/(year × a)	-5.79	2.87	3.02
SD of intercept among years	log odds	0.20		
Probability of maturing as 1SW for males (n = 2891):				
Intercept	log odds	-0.31	0.21	
Contrast to fish caught for broodstock	log odds	0.73	0.22	
Contrast to fish caught in scientific fishing	log odds	0.61	0.21	
Effect of population average smolt age	log odds/year	0.22	0.12	
Effect of population average sea age	log odds/year	-2.26	0.20	
Effect of introgression (log odds/a)	log odds/a	0.29	0.17	1*
SD of intercept among rivers	log odds	0.26		
SD of intercept among years	log odds	0.74		
Probability of maturing as 2SW for females (n = 2175):				
Intercept	log odds	1.10	0.23	
Contrast to fish caught for broodstock	log odds	1.06	0.31	
Contrast to fish caught in scientific fishing	log odds	-0.25	0.30	
Effect of population average smolt age	log odds/year	-0.30	0.21	
Effect of population average sea age	log odds/year	-3.65	0.37	
Effect of introgression (log odds/a)	log odds/a	1.04	0.63	2.75
SD of intercept among rivers	log odds	0.59		
SD of intercept among years	log odds	0.74		
Probability of maturing as 2SW for males (n = 1583):				
Intercept	log odds	1.15	0.24	
Contrast to fish caught for broodstock	log odds	0.26	0.32	
Contrast to fish caught in scientific fishing	log odds	0.05	0.35	
Effect of population average smolt age	log odds/year	-0.16	0.19	
Effect of population average sea age	log odds/year	-2.28	0.34	
Effect of introgression (log odds/a)	log odds/a	0.02	0.26	1**
Interaction: smolt age and introgression	log odds/(year × a)	-3.12	1.71	3.22
SD of intercept among rivers	log odds	0.43		
SD of intercept among years	log odds	0.73		

* The estimated correction factor was negative and therefore ignored (see methods).
** The correction factor was ignored as the original estimate was very close to zero making the correction factor unreasonably large.

Table S4. Parameter estimates for models on size at return and annual growth at sea. Estimates and associated standard error (SE) after correction for attenuation. The correction was done by multiplying the estimate and its standard error by *k*. Sample sizes are given by n. Units are given for each parameter, where "a" is farmed genetic ancestry (i.e. a = 1 is 100% farmed genetic ancestry).

Parameter	Units	Estimate	SE	*k*
Length at age (n = 6227)				
Intercept	ln(mm)	6.34	0.01	
Contrast 2SW	ln(mm)	0.29	0.00	
Contrast 3SW	ln(mm)	0.46	0.00	
Contrast 4SW	ln(mm)	0.54	0.01	
Contrast 5SW	ln(mm)	0.56	0.02	
Contrast to fish caught for broodstock	ln(mm)	0.03	0.01	
Contrast to fish caught in scientific fishing	ln(mm)	-0.02	0.01	
Effect of Julian day	ln(mm)/day	2.5×10^{-4}	5.8×10^{-5}	
Effect of population average smolt age	ln(mm)/year	0.010	0.009	
Effect of population average sea age	ln(mm)/year	0.128	0.012	
Effect of introgression	ln(mm)/a	0.055	0.010	2.09
Interaction: sea age and introgression	ln(mm)/(year × a)	-0.159	0.057	3.47
SD of intercept among rivers	ln(mm)	0.036		
SD of intercept among years	ln(mm)	0.026		
Mass at age (n = 5581)				
Intercept	ln(g)	7.33	0.03	
Contrast 2SW	ln(g)	0.90	0.01	
Contrast 3SW	ln(g)	1.44	0.02	
Contrast 4SW	ln(g)	1.65	0.03	
Contrast 5SW	ln(g)	1.74	0.08	
Contrast to fish caught for broodstock	ln(g)	0.13	0.03	
Contrast to fish caught in scientific fishing	ln(g)	0.08	0.03	
Effect of Julian day	ln(g)/day	7.4×10^{-5}	2.0×10^{-4}	
Effect of population average smolt age	ln(g)/year	0.05	0.03	
Effect of population average sea age	ln(g)/year	0.47	0.05	
Effect of introgression	ln(g)/a	0.12	0.02	1.03
Interaction: sea age and introgression	ln(g)/(year × a)	-0.30	0.11	2.02
SD of intercept among rivers	ln(g)	0.14		
SD of intercept among years	ln(g)	0.08		
Effect of introgression on annual growth at sea (n = 3653)				
Intercept	ln(mm/year)	6.03	0.01	
Contrast 2SW	ln(mm/year)	-0.31	0.01	
Contrast 3SW	ln(mm/year)	-0.49	0.01	
Contrast 4SW	ln(mm/year)	-0.66	0.02	
Contrast 5SW	ln(mm/year)	-0.69	0.11	
Contrast to fish caught for broodstock	ln(mm/year)	0.08	0.04	
Contrast to fish caught in scientific fishing	ln(mm/year)	0.01	0.01	
Effect of Julian day	ln(mm/year)/day	3.6×10^{-4}	0.9×10^{-4}	

Parameter	Units	Estimate	SE	*k*
Effect of population average smolt age	ln(mm/year)/year	0.025	0.011	
Effect of population average sea age	ln(mm/year)/year	0.151	0.016	
Effect of introgression	ln(mm/year)/a	0.065	0.015	1.88
Interaction: smolt age and introgression	ln(mm/year)/(year × a)	-0.032	0.022	1.37
Interaction: sea age and introgression	ln(mm/year)/(year × a)	-0.189	0.093	3.25
SD of intercept among rivers	ln(mm/year)	0.038		
SD of intercept among years	ln(mm/year)	0.031		

* The estimated correction factor was negative and therefore ignored (see methods).

Table S5. Parameter estimates for models on growth at different periods at sea. Estimates and associated standard error (SE) after correction for attenuation. The correction was done by multiplying the estimate and its standard error by *k*. Sample sizes are given by n. Units are given for each parameter, where "a" is farmed genetic ancestry (i.e. a = 1 is 100% farmed genetic ancestry).

Parameter	Units	Estimate	SE	*k*
Growth first year at sea (n = 3700)				
Intercept	ln(mm)	5.68	0.02	
Contrast 2SW	ln(mm)	-0.018	0.007	
Contrast 3SW	ln(mm)	-0.037	0.010	
Contrast 4SW	ln(mm)	-0.046	0.024	
Contrast 5SW	ln(mm)	-0.175	0.148	
Contrast to fish caught for broodstock	ln(mm)	0.070	0.053	
Contrast to fish caught in scientific fishing	ln(mm)	0.026	0.017	
Effect of population average smolt age	ln(mm)/year	-0.004	0.012	
Effect of population average sea age	ln(mm)/year	0.105	0.018	
Effect of introgression	ln(mm)/a	0.052	0.038	3.75
SD of intercept among rivers	ln(mm)	0.041		
SD of intercept among years	ln(mm)	0.095		
Growth second year at sea (n = 2575)				
Intercept	ln(mm)	5.55	0.02	
Contrast 3SW	ln(mm)	0.048	0.009	
Contrast 4SW	ln(mm)	0.006	0.026	
Contrast 5SW	ln(mm)	-0.015	0.168	
Contrast to fish caught in scientific fishing	ln(mm)	0.021	0.023	
Effect of population average smolt age	ln(mm)/year	0.045	0.016	
Effect of population average sea age	ln(mm)/year	0.108	0.029	
Effect of introgression	ln(mm)/a	0.038	0.016	1*
Interaction: sea age and introgression	ln(mm)/(year × a)	-0.125	0.062	1*
SD of intercept among rivers	ln(mm)	0.053		
SD of intercept among years	ln(mm)	0.079		
Growth third year at sea (n = 904)				
Intercept	ln(mm)	5.22	0.03	
Contrast 4SW	ln(mm)	0.095	0.034	
Contrast 5SW	ln(mm)	0.415	0.206	
Contrast to fish caught in scientific fishing	ln(mm)	-0.018	0.040	
Effect of population average smolt age	ln(mm)/year	0.059	0.017	
Effect of population average sea age	ln(mm)/year	0.012	0.036	
Effect of introgression	ln(mm)/a	0.005	0.031	1*
SD of intercept among rivers	ln(mm)	0.021		
SD of intercept among years	ln(mm)	0.080		
Plus growth of 1SW (n = 1106)				
Intercept	ln(mm)	4.83	0.03	
Contrast to fish caught in broodstock fishing	ln(mm)	-0.078	0.118	

Parameter	Units	Estimate	SE	*k*
Contrast to fish caught in scientific fishing	ln(mm)	-0.037	0.058	
Effect of Julian day	ln(mm)/day	2.9×10^{-3}	0.4×10^{-3}	
Effect of population average smolt age	ln(mm)/year	0.095	0.034	
Effect of population average sea age	ln(mm)/year	0.266	0.047	
Effect of introgression	ln(mm)/a	0.111	0.047	1.60
Interaction: smolt age and introgression	ln(mm)/(year × a)	-0.995	0.287	4.42
Interaction: sea age and introgression	ln(mm)/(year × a)	-0.394	0.227	2.22
SD of intercept among rivers	ln(mm)	0.10		
SD of intercept among years	ln(mm)	0.10		
Plus growth of 2SW (n = 1630)				
Intercept	ln(mm)	4.13	0.05	
Contrast to fish caught in scientific fishing	ln(mm)	-0.028	0.075	
Effect of Julian day	ln(mm)/day	2.3×10^{-3}	0.5×10^{-3}	
Effect of population average smolt age	ln(mm)/year	0.117	0.041	
Effect of population average sea age	ln(mm)/year	0.432	0.075	
Effect of introgression	ln(mm)/a	0.518	0.278	5.63
Interaction: sea age and introgression	ln(mm)/(year × a)	-1.91	1.22	3.84
SD of intercept among rivers	ln(mm)	0.33		
SD of intercept among years	ln(mm)	0.16		
Plus growth of 3SW (n = 757)				
Intercept	ln(mm)	3.08	0.09	
Contrast to fish caught in scientific fishing	ln(mm)	0.047	0.156	
Effect of Julian day	ln(mm)/day	2.9×10^{-3}	1.0×10^{-3}	
Effect of population average smolt age	ln(mm)/year	0.131	0.075	
Effect of population average sea age	ln(mm)/year	0.053	0.163	
Effect of introgression	ln(mm)/a	0.037	0.121	1.23
SD of intercept among rivers	ln(mm)	0.16		
SD of intercept among years	ln(mm)	0.22		

* The estimated correction factor was negative and therefore ignored (see methods).

Table S6. Parameter estimates for models on vgll3 and six6. Estimates and associated standard error (SE) after correction for attenuation. The correction was done by multiplying the estimate and its standard error by *k*. Sample sizes are given by n. Units are given for each parameter, where “p” is the allele frequency of the L-allele, “a” is farmed genetic ancestry (i.e. a = 1 is 100% farmed genetic ancestry).

Parameter	Units	Estimate	SE	*k*
vgll3 L-allele frequency (n = 3673)				
Intercept	log odds	-0.777	0.037	
Contrast to fish caught for broodstock	log odds	0.073	0.059	
Contrast to fish caught in scientific fishing	log odds	0.045	0.159	
Effect of population vgll3 allele frequency	log odds/p	4.723	0.188	
Effect of introgression	log odds/a	0.085	0.112	1*
Interaction: vgll3 allele frequency and introgression	log odds/(a × p)	0.950	0.903	1*
SD of intercept among years within rivers	log odds	0.027		
six6 L-allele frequency (n = 3673)				
Intercept	log odds	0.319	0.036	
Contrast to fish caught for broodstock	log odds	0.094	0.062	
Contrast to fish caught in scientific fishing	log odds	0.304	0.164	
Effect of population vgll3 allele frequency	log odds/p	5.025	0.172	
Effect of introgression	log odds/a	-0.020	0.117	1*
Interaction: vgll3 allele frequency and introgression	log odds/(a × p)	-1.148	0.632	1*
SD of intercept among individuals	log odds	0.438		

* The estimated correction factor was less than one and therefore ignored.

Table S7. Overview of data. Columns represent the River ID*, name, number of females (N fem), number of males (N male), number with unknown sex (N sex unkn.) and total number of fish (N total), percentage of data considered representative for late life history (Repr.), percentage from recreational (Recr.), broodstock (Brood.), and scientific fishing (Scient.).

River ID*	River name	N fem	N male	N sex unkn.	N total	Repr.	Recr.	Brood.	Scient.
001.1Z	Enningdalselva	49	70	0	119	92 %	100 %	0 %	0 %
002.Z	Glomma	8	11	0	19	100 %	0 %	100 %	0 %
006.Z	Akerselva	3	2	0	5	0 %	0 %	100 %	0 %
008.Z	Sandvikselva	26	18	1	45	100 %	0 %	100 %	0 %
012.Z	Drammenselva	31	22	3	56	100 %	0 %	100 %	0 %
015.Z	Numedalslågen	36	16	45	97	90 %	80 %	20 %	0 %
016.Z	Skiensvassd.	56	40	13	109	100 %	63 %	37 %	0 %
021.Z	Otra	18	25	15	58	100 %	100 %	0 %	0 %
025.Z	Kvina	13	15	0	28	100 %	100 %	0 %	0 %
027.Z	Bjerkreimselva	58	44	4	106	100 %	0 %	100 %	0 %
028.3Z	Håelva	15	5	0	20	100 %	0 %	100 %	0 %
028.Z	Figgjo	7	5	0	12	0 %	0 %	100 %	0 %
030.2Z	Dirdalselva	8	9	0	17	100 %	0 %	100 %	0 %
033.Z	Årdalselva	49	68	19	136	93 %	78 %	22 %	0 %
035.3Z	Vorma	57	67	39	163	100 %	100 %	0 %	0 %
036.Z	Suldalslågen	71	83	2	156	94 %	90 %	10 %	0 %
037.2Z	Åbøelva	3	2	0	5	0 %	0 %	100 %	0 %
038.Z	Vikedalselva	33	21	0	54	100 %	0 %	0 %	100 %
041.Z	Etneelva	39	44	11	94	100 %	73 %	27 %	0 %
042.3Z	Fjæraelva	3	3	1	7	100 %	0 %	100 %	0 %
044.3Z	Ådlandsvassd	8	5	0	13	100 %	0 %	100 %	0 %
045.4Z	Rosendalselvene	10	8	0	18	100 %	0 %	100 %	0 %
047.2Z	Jondalselva	2	2	1	5	100 %	0 %	100 %	0 %
048.Z	Opo	8	8	1	17	100 %	0 %	100 %	0 %
050.1Z	Kinso	8	6	0	14	100 %	0 %	100 %	0 %
050.Z	Eidfjordvassd.	20	6	2	28	100 %	75 %	25 %	0 %
052.1Z	Granvinselva	3	0	0	3	100 %	0 %	100 %	0 %
052.7Z	Steindalselva	7	6	0	13	100 %	0 %	100 %	0 %
055.7Z	Oselva	6	8	6	20	100 %	100 %	0 %	0 %
060.4Z	Loneelva	44	41	13	98	100 %	70 %	30 %	0 %
061.2Z	Storelva	18	12	0	30	100 %	0 %	100 %	0 %
061.Z	Daleelva	39	25	0	64	100 %	0 %	100 %	0 %
062.Z	Vosso	44	47	0	91	100 %	12 %	3 %	85 %
070.Z	Vikja	15	6	2	23	100 %	0 %	100 %	0 %
072.2Z	Flåmselva	5	6	0	11	100 %	0 %	100 %	0 %
073.Z	Lærdalselva	91	118	22	231	65 %	100 %	0 %	0 %
075.Z	Fortunselva	2	8	0	10	100 %	0 %	100 %	0 %
077.Z	Årøyelva	73	56	4	133	73 %	80 %	20 %	0 %
079.Z	Daleelva	127	91	4	222	97 %	97 %	3 %	0 %
082.5Z	Storelva	31	32	17	80	100 %	100 %	0 %	0 %

River ID*	River name	N fem	N male	N sex unkn.	N total	Repr.	Recr.	Brood.	Scient.
082.Z	Flekkeelva	60	51	5	116	85 %	94 %	6 %	0 %
083.2Z	Kvamselva	4	4	0	8	0 %	0 %	100 %	0 %
083.Z	Gaula	15	6	0	21	0 %	0 %	100 %	0 %
084.7Z	Nausta	77	34	16	127	100 %	66 %	34 %	0 %
084.Z	Jølstra	63	54	10	127	100 %	76 %	20 %	3 %
085.Z	Osenelva	24	13	0	37	100 %	0 %	100 %	0 %
087.1Z	Ryggelva	34	47	28	109	100 %	100 %	0 %	0 %
087.Z	Gloppenelva	53	53	2	108	100 %	100 %	0 %	0 %
088.1Z	Oldenelva	13	8	8	29	100 %	100 %	0 %	0 %
088.Z	Strynselva	44	23	0	67	100 %	100 %	0 %	0 %
089.4Z	Hjalma	46	57	3	106	100 %	100 %	0 %	0 %
089.Z	Eidselva	51	45	14	110	100 %	100 %	0 %	0 %
092.Z	Åheimselva	8	8	12	28	100 %	100 %	0 %	0 %
093.2Z	Oselva	14	3	0	17	100 %	0 %	100 %	0 %
095.3Z	Vartdalselv	27	61	12	100	100 %	100 %	0 %	0 %
095.Z	Ørstaelva	14	5	0	19	0 %	0 %	100 %	0 %
097.2Z	Vikelva	10	8	0	18	100 %	100 %	0 %	0 %
097.72Z	Aureelva	9	6	0	15	0 %	0 %	100 %	0 %
097.7Z	Velledalselva	16	13	0	29	100 %	0 %	100 %	0 %
101.2Z	Solnørelva	23	18	1	42	100 %	100 %	0 %	0 %
101.6Z	Tennfjordelva	6	12	12	30	100 %	100 %	0 %	0 %
102.6Z	Tressa	48	15	7	70	100 %	100 %	0 %	0 %
103.1Z	Måna	29	13	9	51	100 %	100 %	0 %	0 %
104.2Z	Visa	2	1	0	3	100 %	100 %	0 %	0 %
104.Z	Eira	124	149	8	281	52 %	93 %	7 %	0 %
105.Z	Oselva	71	49	27	147	100 %	69 %	0 %	31 %
107.3Z	Sylteelva	34	34	51	119	100 %	100 %	0 %	0 %
109.Z	Driva	46	75	66	187	100 %	71 %	0 %	29 %
111.7Z	Søya	9	9	2	20	100 %	100 %	0 %	0 %
111.Z	Todalselva	22	13	2	37	100 %	54 %	46 %	0 %
112.3Z	Bævra	25	15	6	46	100 %	70 %	30 %	0 %
112.Z	Surna	161	439	70	670	99 %	91 %	6 %	3 %
121.Z	Orkla	9	45	4	58	100 %	100 %	0 %	0 %
122.1Z	Børselva	18	8	1	27	100 %	52 %	0 %	48 %
122.2Z	Vigda	36	12	5	53	100 %	100 %	0 %	0 %
122.Z	Gaulavassd	25	34	6	65	92 %	92 %	8 %	0 %
123.4Z	Homla	34	20	15	69	100 %	100 %	0 %	0 %
123.Z	Nidelva	56	43	1	100	100 %	100 %	0 %	0 %
124.Z	Stjørdalsvassd.	9	1	0	10	0 %	0 %	100 %	0 %
126.6Z	Levangerelva	5	1	0	6	100 %	100 %	0 %	0 %
128.Z	Steinkjervassd.	11	10	0	21	100 %	0 %	100 %	0 %
132.Z	Skauga	20	14	0	34	100 %	0 %	0 %	100 %
133.3Z	Nordelva	13	22	10	45	100 %	100 %	0 %	0 %

River ID*	River name	N fem	N male	N sex unkn.	N total	Repr.	Recr.	Brood.	Scient.
134.Z	Teksdalselva	30	19	6	55	100 %	100 %	0 %	0 %
135.Z	Norddalselva	9	7	5	21	100 %	100 %	0 %	0 %
137.2Z	Steinsdalselva	55	36	8	99	100 %	63 %	0 %	37 %
138.5Z	Aursunda	0	0	19	19	100 %	100 %	0 %	0 %
138.6Z	Bogna	30	8	49	87	100 %	11 %	89 %	0 %
138.Z	Årgårdsvassd.	8	6	6	20	100 %	100 %	0 %	0 %
139.Z	Namsen	21	55	60	136	93 %	85 %	0 %	15 %
144.Z	Åelva	46	39	12	97	100 %	100 %	0 %	0 %
159.21Z	Gjervalelva	8	10	1	19	100 %	100 %	0 %	0 %
160.43Z	Reipåvassd.	12	16	1	29	100 %	100 %	0 %	0 %
161.Z	Beiarvassd.	64	86	4	154	97 %	63 %	37 %	0 %
163.Z	Saltdalsvassd.	16	80	2	98	100 %	99 %	0 %	1 %
172.Z	Forsåvassd.	16	4	0	20	100 %	100 %	0 %	0 %
173.Z	Skjoma	37	26	1	64	98 %	73 %	0 %	27 %
174.5Z	Elvegårdselva	13	14	0	27	70 %	100 %	0 %	0 %
175.4Z	Tårstadvassd.	17	16	2	35	100 %	100 %	0 %	0 %
185.1Z	Alsvågvassd.	16	20	2	38	100 %	100 %	0 %	0 %
186.2Z	Roksdalsvassd.	5	4	11	20	100 %	100 %	0 %	0 %
186.62Z	Bleikvassd.	3	0	1	4	0 %	0 %	100 %	0 %
189.3Z	Renså	1	2	0	3	100 %	0 %	0 %	100 %
191.Z	Salangsvassd.	12	33	4	49	100 %	100 %	0 %	0 %
194.Z	Laukhelle	8	3	9	20	100 %	100 %	0 %	0 %

*Unique code for Norwegian watercourse outlets issued by The Norwegian Water Resource and Energy Directorate (NVE).
